# The genus *Scutellathous* Kishii, 1955 (Coleoptera, Elateridae, Dendrometrinae) in China, with description of three new species

**DOI:** 10.3897/zookeys.857.29011

**Published:** 2019-06-25

**Authors:** Zhen Liu, Shi-hong Jiang

**Affiliations:** 1 School of Applied Chemistry and Biological Technology, Postdoctoral Innovation Practice Base, Shenzhen Polytechnic, Shenzhen, Guangdong 518055, China; 2 College of life and environmental sciences, Hunan University of Arts and Science, Changde, Hunan 415000, China; 3 College of Natural Resources and Environment, South China Agricultural University, Guangzhou, Guangdong 510642, China

**Keywords:** *
Athous
*, catalogue, distribution, Elateroidea, key, new taxa, *
Ohirathous
*

## Abstract

Five species of *Scutellathous* Kishii, 1955 are recognized from China, of which three are new. *Scutellathoushabenularis***sp. nov.**, *S.nanlingensis***sp. nov.**, and *S.quadrata***sp. nov**. are described and illustrated. A key to species from China, a checklist, and a distribution map are provided. The relationships and comparisons among genera *Athous* Eschscholtz, 1829, *Ohirathous* Han & Park, 2012, *Parathous* Fleutiaux, 1918, and *Scutellathous* Kishii, 1955 are discussed.

## Introduction

The genus *Scutellathous* (Coleoptera, Elateridae), as currently defined, is distributed only in East Asia: China (Taiwan), Japan, and Korea. It was erected by Kishii (1955) based on the type species, *Athouscomes* Lewis, 1894 from Japan. Kishii (1955) also transferred *S.porrecticollis* (Lewis, 1894) to this genus and described *S.horior*. The latter has been moved to the genus *Stenagostus* Thomson, 1959 (Kishii 2001). Later, eight species were described in *Scutellathous*: *S.fujianus* Ôhira, 1963, *S.ozakii* Ôhira, 1992, *S.sasajii* Kishii, 2001, *S.seinoi* Kishii, 2001, *S.shikokuanus* Kishii, 1985, and *S.yakuensis* Nakane & Kishii, 1958 from Japan; *S.spinosus* Platia & Schimmel, 2007 and *S.yamashitai* Arimoto, 1992 from China (Taiwan). There are currently, 10 species recognized within the genus.

Ôhira (1996) synonymized *Scutellathous* under genus *Parathous* Fleutiaux, 1918, known from south-west Asia, based on similarity of the male genitalia, which have simple parameres apices. Kishii (2001) reviewed the Japanese species of *Scutellathous* and argued for genus status because of consistent differences from *Parathous* in the supra-antennal carina, supra-orbital groove, pronotum, pronotal hind angles and elytra.

*Scutellathous* includes mid-sized beetles (body length: 11–16 mm), resembling the monotypic genus *Ohirathous* Han & Park, 2012 in sharing similar shaped frons (supra-antennal carina thickened, overhanging nasale, raised above part of frons immediately posterior to it, (‘pentroof shape’, *sensu*Kishii 2001)) and broad sub-lateral incisions (Han et al. 2012), but with shorter carinae on hind angles (extending only 1/5 to 1/3 length of pronotum; reaching to 1/2 length in *Ohirathous*), the proportions of antennomere 3 to 2 (antennomere 3 usually over twice longer than 2; it is 1.8 times in *O.nantouensis*), the apical lobed apices of tarsomeres 2 and 3 (*O.nantouensis* lobed from 1^st^ to 4^th^ tarsomeres), the shape of female pronotum (arched laterally, narrowed anteriorly in dorsal view; parallel-sided in *O.nantouensis*) and other characters (Han et al. 2012, 2016).

During our study of the Chinese elaterids, we found three undescribed species from south China. These new species resemble monotypic genus *Ohirathous* Han & Park, 2012 in sharing the ‘pentroof’ shape of frons and broad sublateral incisions (Han et al. 2012), but should be placed in *Scutellathous* because of its male genitalia (aedeagus with simple, narrow paramere apices, without apico-lateral expansion at apex, penis gradually narrowed and acute at apex) and the other diagnostic characters mentioned above, and conforming to the generic diagnosis of Han et al. (2016).

## Material and methods

The studied specimens are deposited in the following collections:

**SZPT**School of Applied Chemistry and Biological Technology, Shenzhen Polytechnic, Shenzhen, Guangdong Province, China.

**MHBU** Museum of Hebei University, Baoding, China.

The terminology used mainly follows Costa et al. (2010), Douglas (2011). The classification follows Cate et al. (2007). Descriptions and measurements were made under a stereomicroscope (Motic SMZ-168). Photographs of types were taken using a digital microscope (LY-WN-YH 3D system) and a Canon 800D camera with a Canon EF 65 mm lens. The genitalia of holotypes and paratypes were macerated in 10% NaOH and photographed in a glycerin jelly.

Measurements: body length was measured along the midline from the anterior edge of the head capsule to the apex of the elytra; the body width was measured across the broadest part (usually across the elytra). The pronotal length was measured along the midline; the pronotal width was measured at the broadest part (usually at the hind angles). The ocular index is obtained by dividing the minimum distance between the eyes by the maximum distance across both eyes and multiplying the quotient by 100 (Becker 1979).

The specimens were mounted on paper points. The genitalia were removed, cleaned and fixed under the body of the specimen in glycerol mounts as described by Prosvirov and Savitsky (2011).

The studied specimens were all collected by hand netting.

## Taxonomy

### Scutellathous

Taxon classificationAnimaliaColeopteraElateridae

Kishii, 1955

Scutellathous Kishii, 1955: 79 (type species: Athouscomes Lewis, 1894: 200 (Sapporo, Japan; by original designation); Ôhira 1970: 22; Gurjeva 1974: 108; Kishii 1987: 91; Park et al. 1993: 179; Suzuki 1999: 113; Kishii 2001: 206; Cate et al. 2007: 172; Han et al. 2016: 72.

#### Diagnosis.

Body length: 11–16 mm; frons triangularly depressed behind supra antennal carina, which is strongly thickened and overhanging labrum and nasale, carina elevated above part of frons immediately posterior to it in dorsal view; supra-orbital groove broadly excavated; antennae serrate from 3^rd^ antennomere; pronotum mostly longer than wide, pronotal disc with weak median depression; hind angles of pronotum unicarinate; sublateral incisions at posterior margin of pronotum small, or tooth-like; prosternal sutures not grooved anteriorly; apical end of tarsomeres 2 and 3 lobed beneath; aedeagus with simple and narrow parameres apices, without apico-lateral expansion at apex, penis gradually narrowed and acute at apex (after Han et al. 2016).

#### Distribution.

China, Japan, and Korea.

#### Remarks.

Based on a study of the descriptions and photos of *Athous* (Elateridae, Dendrometrinae) species from North America, we found the *Athouscucullatus* (Say, 1825) species-group shares many characters with *Scutellathous* (Becker, 1979). These include head flattened with triangular depression; frontal carina prominent, well elevated above labrum; eyes large; punctures on pronotum umbillicate; tarsomeres 2- and 3-lobed; and male genitalia with parameres lacking subapical lateral tooth and dorsal carina. However, lobes on tarsomeres 2 and 3 are smaller in *Scutellathous*, the carinae on the pronotal hind angles are present and sharp in all *Scutellathous* (absent or present in *A.cucullatus* group), and base of pronotum with sublateral incisions near hind angles in all *Scutellathous* (some without incisions in *A.cucullatus* group). Further comparison of members of the North American *Athouscucullatus* species group to *Ohirathous* is needed.

##### Key to the male species of *Scutellathous* Kishii from China

**Table:** 

1	Pronotum with a median furrow (Fig. 7b) or non-furrowed glabrous line (Figs 2e, 9a) through the entire length	**2**
–	Pronotum with a median furrow only on posterior half, never with non-furrowed glabrous line	**4**
2	Aedeagus with penis reaching beyond parameres (Fig. 8a–d); scutellar shield 1.5 times longer than wide (Fig. 7f)	***S.nanlingensis* sp. nov.**
–	Penis reaching only to apex of parameres (Figs 4a–c, 11e); scutellar shield 1.1 times longer than wide (Figs 2f, 11b)	**3**
3	More pubescent, dorsal pubescence partially hiding integument (Fig. 9a); body brown-black; pronotal hind angles convergent posterad (Fig. 10c)	***S.quadrata* sp. nov.**
–	Less pubescent and more shiny (Fig. 1a); body red-brown; pronotal hind angles divergent posterad (Fig. 2e)	***S.habenularis* sp. nov.**
4	Elytra with a short spine near humeral angles in dorsal view; antenna falling short of pronotal hind angle apex by length of the last antennomere; hind angles not divergent posterad	***S.spinosus* Platia & Schimmel, 2007**
–	Elytra without spine near humeral angles; antenna longer, exceeding apex of hind angle of pronotum by at least length of two antennomeres; hind angles divergent	***S.yamashitai* Arimoto, 1992**

##### Checklist of *Scutellathous* from China

*Scutellathoushabenularis* sp. nov. [China (Yunnan)]

*Scutellathousnanlingensis* sp. nov. [China (Guangdong)]

*Scutellathousquadrata* sp. nov. [China (Zhejiang)]

*Scutellathousspinosus* Platia & Schimmel, 2007 [China (Taiwan)]

*Scutellathousyamashitai* Arimoto, 1992 [China (Taiwan)]

### Scutellathous habenularis

Taxon classificationAnimaliaColeopteraElateridae

Liu & Jiang sp. nov.

http://zoobank.org/FBAA77C9-3CBF-4DED-812E-98F9E3D13910

**Figure 1.**
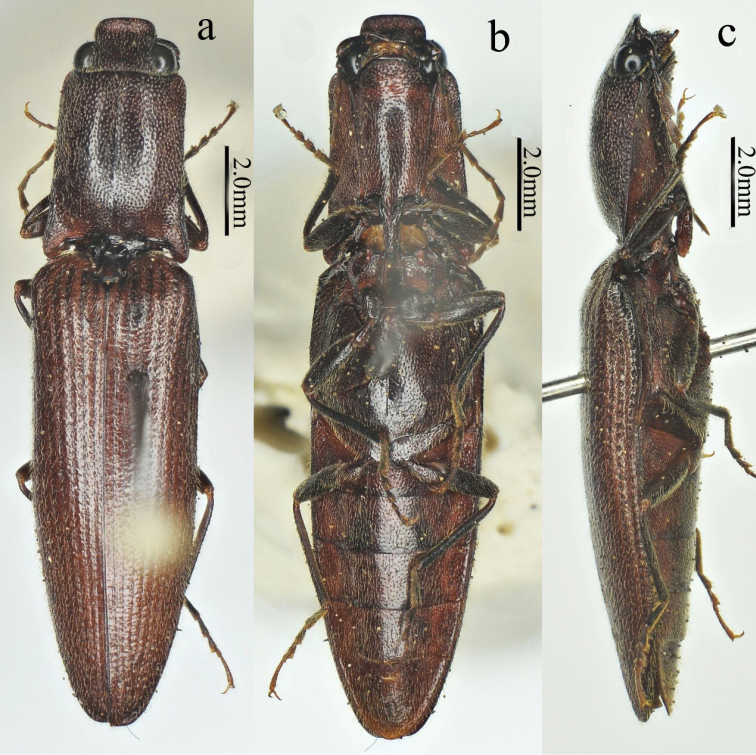
Habitus of *Scutellathoushabenularis* sp. nov., holotype, male **a** dorsal view **b** ventral view **c** lateral view.

**Figure 2.**
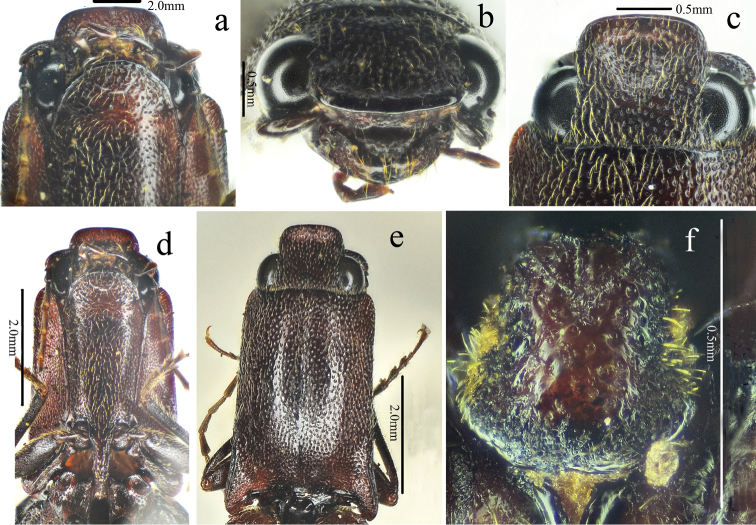
*Scutellathoushabenularis* sp. nov., holotype, male **a** head, ventral view **b** head, antero-dorsal view **c** head, dorsal view **d** prothorax, ventral view **e** pronotum, dorsal view **f** scutellar shield, dorsal view.

**Figure 3.**
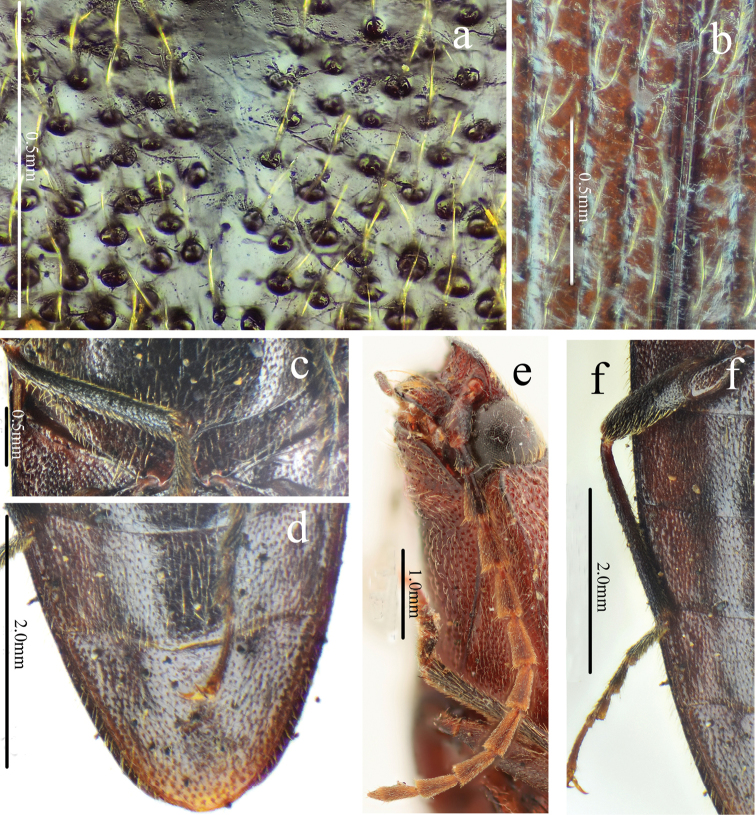
*Scutellathoushabenularis* sp. nov., holotype, male surface of pronotum, dorsal view **b** surface of elytra, dorsal view **c** hind coxae, ventral view **d** sternite VII, ventral view **e** antenna, lateral view **f** hind leg, anterior view.

**Figure 4.**
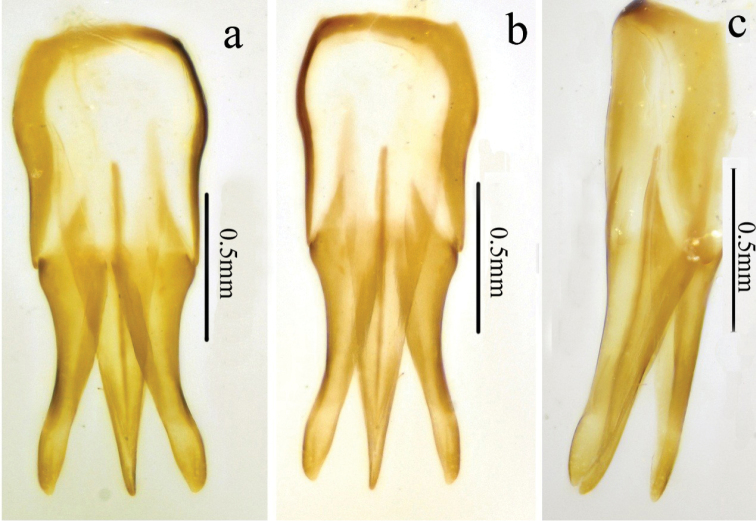
Aedeagus of *Scutellathoushabenularis* sp. nov., holotype, male **a** dorsal view **b** ventral view **c** lateral view.

**Figure 5.**
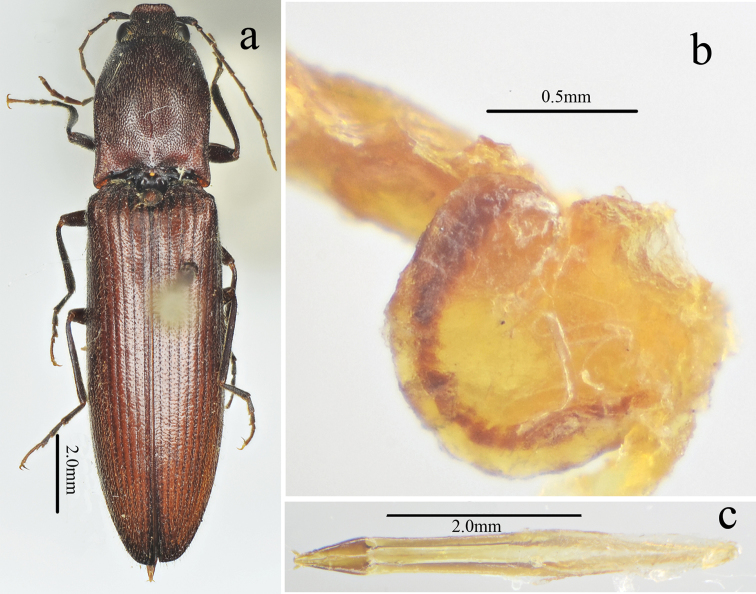
*Scutellathoushabenularis* sp. nov., paratype, female **a** habitus, dorsal view **b** bursa copulatrix (before dissolved macerating in 10% NaOH for 10 hours) **c** ovipositor, dorsal view.

Figs 1 , 2 , 3 , 4 , 5 , 12

#### Type locality.

Yunnan, China.

#### Material examined.

Holotype: ♂ (MHBU), labels: 1) Yunnan Prov., Gaoligong Mts (2000 m, 25°59'8.81"N 98°49'1.40"E) (高黎贡山), 2012.VII.23, leg. Ji-shan Xu et Ling-xiao Chang, Shenzhen Polytechnic; 2) Holotype, *Scutellathoushabenularis* sp. nov., Liu et al. 2019; 3) No. 20180353. Paratype: 1♀ (MHBU), labels: 1) Yunnan Prov., Gaoligong Mts (2000 m, 25°59'8.81"N 98°49'1.40"E) (高黎贡山), 2012.VII.23, leg. Ji-shan Xu et Ling-xiao Chang, Shenzhen Polytechnic; 2) Paratype, *Scutellathoushabenularis* sp.nov., Liu et al. 2019; 3) No. 20180354.

#### Diagnosis.

Body bright red-brown, shiny; anterior edge of head truncate in dorsal view; antennae reaching apices of pronotal hind angles, nearly cylindrical from antennomere 6 onward, attached apico-dorsally, antennomere 3 1.8 times as long as 2 and nearly as long as 4; pronotum with hind angles divergent posterad, disc flat medially with only trace of a glabrous non-furrowed longitudinal line, shiny with umbillicate punctures; scutellar shield 1.1 times longer than wide; aedeagus with penis reaching to apex of parameres, penis gradually narrowed to pointed apex.

*Scutellathoushabenularis* is similar to *S.yamashitai*Arimoto 1992, but is distinguished by the glabrous non-furrowed longitudinal line throughout the length of the pronotum (with shallow median impression only on basal half of the pronotum in *S.yamashitai*), the obtuse anterior angles of the pronotum (acute in *S.yamashitai*), and the penis reaching beyond parameres (penis shorter than parameres in *S.yamashitai*).

#### Description.

Male (holotype). Body (Fig. 1a–c) length 14.8 mm, width 3.5 mm; bright red-brown on head, pronotum (except hind angles), and elytra near scutellar shield; ventral surfaces and legs brown-black, and antennae brown (except antennomere 1–2 brown-black); dorsal pubescence yellow, suberect, shorter, denser, and pointed anterad on pronotum and head, longer, sparser, semi-recumbant, pointed posterad on elytra, ventral pubescence more recumbent and thinner.

*Head.* Anterior edge truncate in dorsal view (Fig. 2e), spaces between punctures shiny and 1–2 puncture diameters wide, punctures larger, denser, coarser in deep triangular depression (Fig. 2c); eye semi-spherical, ocular index 71; last segment of maxillary palpus 1.6 times longer than wide; antennae (Fig. 3e), reaching apices of pronotal hind angles, antennomeres 3 to 10 weakly serrate, nearly cylindrical from antennomere 6 onward, attached apico-dorsally, antennomere 1 robust, longest and subclavate, antennomere 2 shortest, obconic, 1.4 times longer than wide, antennomere 3 elongated triangular, 1.8 times as long as 2 and nearly as long as 4, antennomere 11 oblong, 5.0 times longer than wide, 1.1 times longer than antennomere 3, proportions of antennomeres as follows: 100; 52; 93; 95; 95; 94; 82; 96; 87; 82; 98.

*Thorax.* Pronotum (Fig. 2e) nearly 1.2 times longer than wide, parallel-sided in dorsal view, except abruptly concave at anterior end and strongly concave before hind angles, widest at hind angles; disc flat medially with only trace of a glabrous non-furrowed longitudinal line, shiny with umbillicate punctures, spaces between punctures 1 to 2 puncture diameters wide medially (Fig. 3a), strongly umbillicate with interspaces 0.3 puncture diameter wide laterally and posteriorly; hypomera (Fig. 2d) with spaces between punctures narrower than half puncture diameter wide; hind angles (Fig. 2e) divergent, apex upheaved and obtuse, with carina extending only to basal 1/5 of pronotum along sides; sublateral incisions small. Prosternum shiny and sparsely, irregularly punctate, interspaces 1 to 4 times puncture diameters wide; anterior lobe (Fig. 2a) 2.3 times wider than long, with sparse, fine punctures. Prosternal process gradually narrowed to pointed apex. Meso- and meta-ventrites with stronger and denser punctures than those on prosternum, smooth. Metaventrite furrowed medially on anterior 3/4.

*Scutellar shield.* 1.1 times longer than wide (Fig. 2f), widest posterad, narrowing anterad gradually at sides, hind margin arched, weakly emarginate anteriorly; disc convex with umbillicate punctures medially, spaces between punctures 2–3 puncture diameters wide, strongly rugose-punctate near edges, with short yellow, outwardly-oriented pubescence laterally.

*Elytra.* Slender (Fig. 1a), 2.7 times longer than wide, 2.8 times longer and 1.1 times wider than prothorax respectively, widest at anterior one-fifth, parallel-sided, gradually narrowing to apex from midlength (Fig. 3b) with punctate striae, interpuncture spaces about 2–3 puncture diameters wide, interstriae slightly elevated, mostly smooth with smaller, shallower and sparser punctures than punctures within striae, interpuncture spaces about 3 puncture diameters wide, weakly and transversely rugulose anteriorly.

*Legs.* Slender (Fig. 3f); tarsomere 3 with a larger lobe beneath than tarsomeres 2, tarsomere 4 shortest; metacoxal plate (Fig. 3c) with mesal parts subparallel-sided, then abruptly strongly narrowed into at lateral one-fourth.

*Abdomen.* Surfaces of sternites III–VII like metaventrite, with punctures and pubescence more regular and evenly distributed laterally; sternite VII (Fig. 3d) broadly rounded, elongate, 1.7 times wider than long, punctures becoming bigger posteriorly.

*Genitalia.* Aedeagus (Fig. 4a–c) slender, weakly sclerotized; penis reaching to apex of parameres, gradually narrowed to pointed apex; parameres thin, strongly sinuate laterally at midlength, tapered to rounded apex.

**Female.** Like male (Fig. 5a), except larger (length: 15.9 mm, width: 3.9 mm), antennae shorter (short of pronotal hind angle apices by half length of last antennomere) and pronotum strongly arched laterally and narrowed anteriorly in dorsal view. Bursa copulatrix (Fig. 5b) weakly sclerotized (it dissolved after being macerated in 10% NaOH for 10 hours) with a circular thorny line and without thorny plates. Ovipositor (Fig. 5c) 2.3 times longer than wide, with short styli.

**Larva.** Unknown.

#### Etymology.

The name of the new species is derived from the Latin “*habenularis*” (Latin for “strip”) referring to its hind coxae abruptly and strongly narrowed into a strip at lateral one-fourth part.

#### Distribution.

China: Yunnan (Fig. 12).

#### Biology.

Collected around the elevation of 2000 m in subtropical evergreen forest.

#### Remarks.

Its bursa copulatrix resembles *Ohirathous* in sharing a circular thorny line, but differs in lacking thorny plates. Other characters (carina on hind angles extending only basal 1/5 of pronotal length; antennomere 3 2.1 times as long as 2; shape of male genitalia; shape of female pronotum; only tarsomeres 2 and 3 lobed beneath) are consistent with the diagnosis of genus *Scutellathous*. Structures of the bursa copulatrix are diverse within *Scutellathous* species – from five thorny plates (*S.comes*, *S.porrecticollis*, *S.sasajii*, *S.shikokuanus*) or four (*S.seinoi*) thorny plates and without thorny line to no thorny plates but with thorny line in *S.habenularis*. The bursa copulatrix structure of *Ohirathous* (a circular thorny line and two thorny plates) falls within the range of variation observed in *Scutellathous* and does not support distinction of *Ohirathous* from *Scutellathous*. Unfortunately, male genitalia of *Ohirathous* is unknown. Further phylogenetic study is needed to understand the monophyly of *Ohirathous* and the relationship between these two genera.

### Scutellathous nanlingensis

Taxon classificationAnimaliaColeopteraElateridae

Liu & Jiang sp. nov.

http://zoobank.org/25E7432B-DB2B-42CB-916A-50C7E0DCA4A5

Figs 6 , 7 , 8 , 12

#### Type locality.

Ruyuan Nanling Mts, Guangdong, China.

#### Material examined.

Holotype: ♂ (SZPT), labels: 1) Guangdong Prov., Ruyuan Nanling Mts (961 m, 24°55'31.02"N 113°01'18.33"E) (南岭), 2008.VI–VII, leg. Lei Gao et Kai-xuan Chen; 2) Holotype, *Scutellathousnanlingensis* sp.nov., Liu et al. 2019; 3) No. 20180355.

#### Diagnosis.

Antennae barely reaching apices of pronotal hind angles, antennomere 3 2.2 times longer than 2 and 1.1 times longer than 4; pronotum with narrow median furrow throughout length, shiny with fine, weakly umbillicate punctures; scutellar shield 1.5 times longer than wide; punctures on elytra becoming absent near apex; aedeagus with penis reaching beyond parameres, gradually narrowing to obtuse apex.

*Scutellathousnanlingensis* is similar to *S.sasajii* Kishii, 2001, but can be separated from the latter by the short antennae of male (hardly or just reaching apices of pronotal hind angles; longer in *S.sasajii*, with apical three antennomeres exceeding apices of the hind angles), with narrow median furrow through the length of the pronotum (in *S.sasajii* the pronotum lacks longitudinal furrow), and the longer male penis.

#### Description.

**Figure 6.**
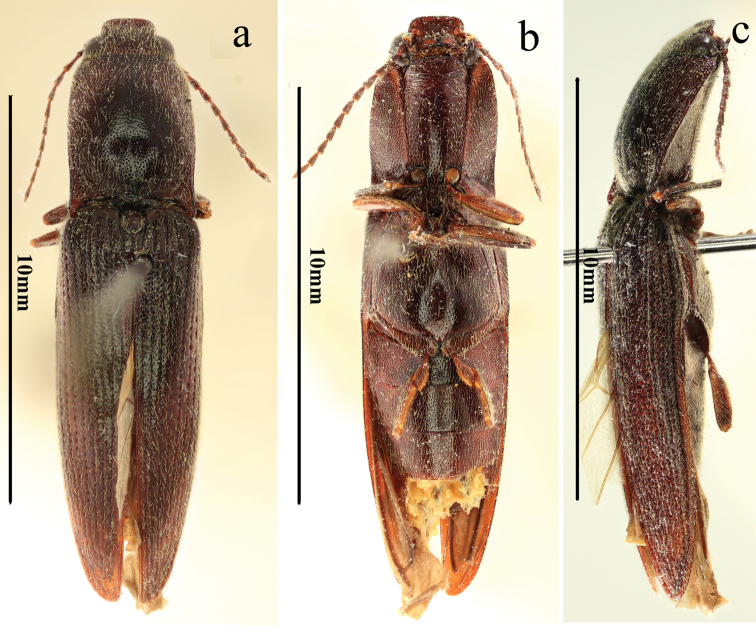
Habitus of *Scutellathousnanlingensis* sp. nov., holotype, male **a** dorsal view **b** ventral view **c** lateral view.

Male (holotype). Body (Fig. 6a–c) length 11.7 mm, body width 3.0 mm; red-brown, head, pronotum, base of elytra, ventral parts of the body and antennae darker, apex of mandible, inner margin of hypomeron, fore and middle coxae, apical parts of legs and elytra paler, more yellowish or reddish; dorsal pubescence yellow, semi-recumbent, longer, sparse and pointed anterad on pronotum and head, pointed posterad on elytra, ventral pubescence more recumbent, thinner and denser, especially on abdomen.

**Figure 7.**
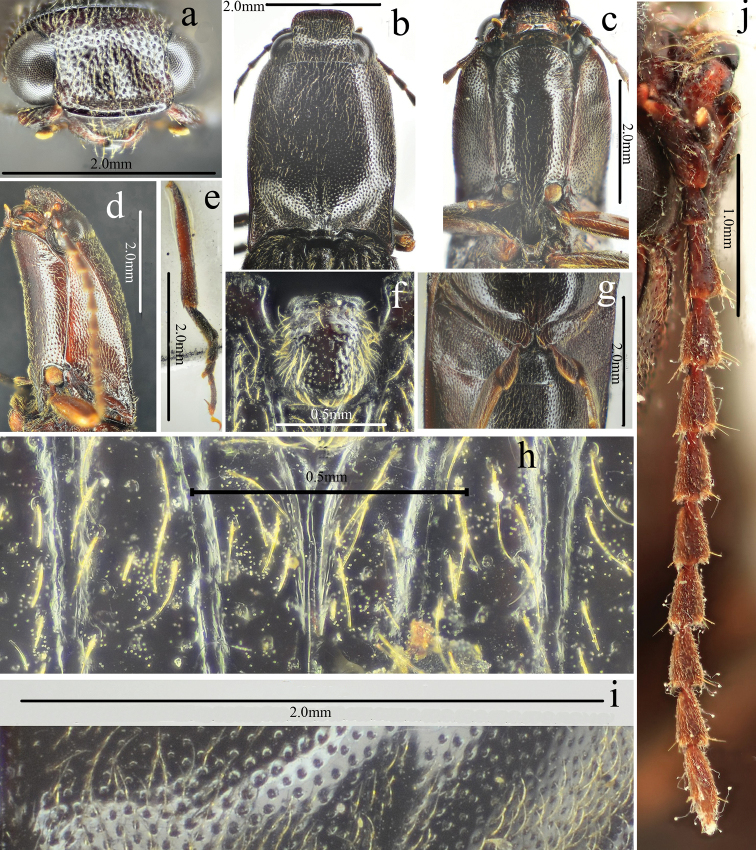
*Scutellathousnanlingensis* sp. nov., holotype, male **a** head, antero-dorsal view **b** pronotum, dorsal view **c** prothorax, ventral view **d** prothorax, lateral view **e** middle leg, dorso-lateral view **f** scutellar shield, dorsal view **g** hind coxae, ventral view **h** surface of elytra, dorsal view **i** surface of pronotum, dorsal view **j** antenna, lateral view.

*Head.* Anterior edge truncate in dorsal view (Fig. 7b), spaces between punctures shiny and 1 puncture diameter wide, punctures umbillicate and uniform, nearly contiguous in triangular, shallow depression and near supra antennal carina (Fig. 7a); eye, semi-spherical, ocular index 74; last segment of maxillary palpus 2.5 times longer than wide; antennae (Figs 6a, 7j) barely reaching apices of pronotal hind angles, antennomere 3 to 10 moderately serrate, attached apico-dorsally, antennomere 1 robust, longest and subclavate, antennomere 2 shortest, obconic, 1.7 times longer than wide, antennomere 3 elongated triangular, 2.2 times longer than 2 and 1.1 times longer than 4, antennomere 11 oblong, 5.6 times longer than wide, 1.1 times longer than antennomere 3, proportions of antennomeres as follows: 100; 43; 93; 82; 80; 76; 75; 70; 67; 72; 99.

*Thorax.* Pronotum (Fig. 7b) nearly 1.2 times longer than wide in dorsal view, parallel-sided, except strongly arched anteriorly and weakly concave before hind angles, widest at posterior 1/3 and apices of hind angles; disc broadly flat medially, with narrow median furrow throughout length (Fig. 7b), shiny with fine, weakly-umbillicate punctures, spaces between punctures (Fig. 7i) 1 to 2 puncture diameters wide medially, weakly umbillicate with interspaces 0.5 to 1 puncture diameter wide laterally and posteriorly; hypomera with spaces between punctures 1 to 2 puncture diameters wide; hind angles not divergent, apex acute, with carina reaching basal third of pronotum, gradually approaching side posterad; sublateral incisions long. Prosternum (Fig. 7c, 7d) shiny and sparsely punctate, interspaces 2 to 3 puncture diameters wide; anterior lobe 2.4 times wider than long, with dense, coarse, punctures. Prosternal process (Fig. 7c, 7d) straight in lateral view, abruptly concave behind procoxae and obtusely pointed at apex. Meso- and meta-ventrites smooth with punctures like on prosternum. Metaventrite furrowed medially throughout length, except shallow to absent on the posterior 1/10.

*Scutellar shield.* (Fig. 7f) 1.5 times longer than wide, parallel-sided, weakly pointed anteriorly, posterior edge arched; disc convex, with small, simple, sparse punctures, spaces between punctures 2–3 puncture diameter wide, with long, yellow outwardly-oriented pubescence.

*Elytra.* Slender, 2.8 times longer than wide, 2.8 times longer and 1.2 times wider than prothorax, longitudinally oviform, shiny, anterior half nearly parallel-sided, narrowing to apex from midlength, widest at apical third, with deeply-punctate striae, strial punctures elongate, the interpuncture spaces about 1–2 puncture diameters wide (Fig. 7h), the interstriae elevated, smooth with small irregular and sparser punctures, interpuncture spaces about 2 to 3 puncture diameters wide, punctures becoming absent near apex (Fig. 6a).

*Legs.* Slender (Fig. 7e); tarsomere 3 with longer lobe than tarsomere 2, tarsomere 4 shortest; metacoxal plate (Fig. 7g) with basal half parts subparallel-sided, then gradually narrowing laterally.

*Abdomen.* Surface (Fig. 6b, 7g) of sternites III-VII like metaventrite, with punctures denser and pubescence shorter; sternites VI–VII missing.

**Figure 8.**
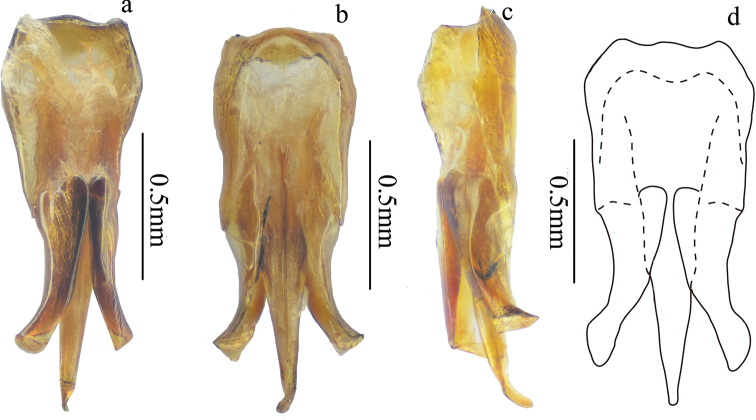
Aedeagus of *Scutellathousnanlingensis* sp. nov., holotype **a** dorsal view **b** ventral view **c** lateral view **d** dorsal view (not shrunken).

*Genitalia.* Aedeagus (Fig. 8a-d) with penis reaching beyond parameres, gradually narrowing to obtuse apex; parameres sinuate laterally at midlength, narrowed before rounded pre-apical expansion, apices rounded-acute.

**Female.** Unknown.

**Larva.** Unknown.

#### Etymology.

The new species named after Nanling Mts in Guangdong prov., referring to its only known locality.

#### Distribution.

China: Guangdong (Nanling Mts) (Fig. 12).

#### Biology.

Unknown.

#### Remarks.

Genitalia of this specimen is shrunken, membranous parameres apices are folded in (Fig. 8c).

### Scutellathous quadrata

Taxon classificationAnimaliaColeopteraElateridae

Liu & Jiang sp. nov.

http://zoobank.org/FAD53D97-CBB9-439D-A0B3-C509DB413292

**Figure 9.**
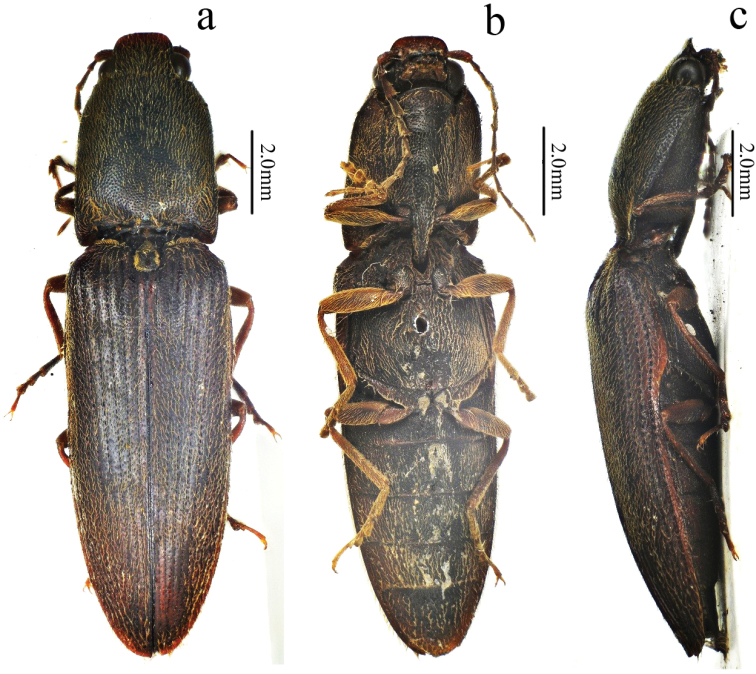
Habitus of *Scutellathousquadrata* sp. nov., holotype, male **a** dorsal view **b** ventral view **c** lateral view.

**Figure 10.**
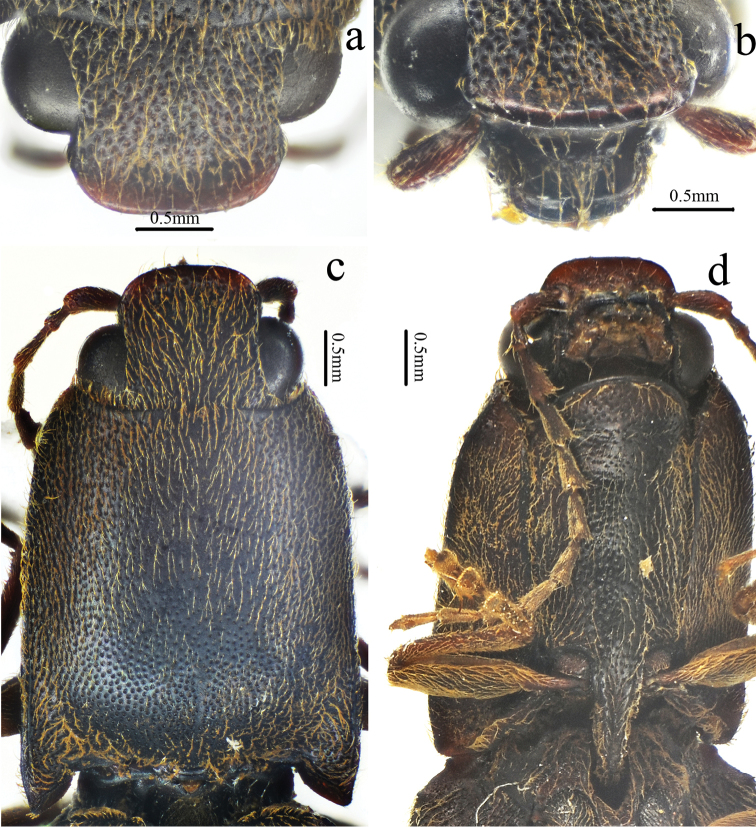
*Scutellathousquadrata* sp. nov., holotype, male **a** head, dorsal view **b** head, antero-dorsal view **c** pronotum, dorsal view **d** prothorax, ventral view.

**Figure 11.**
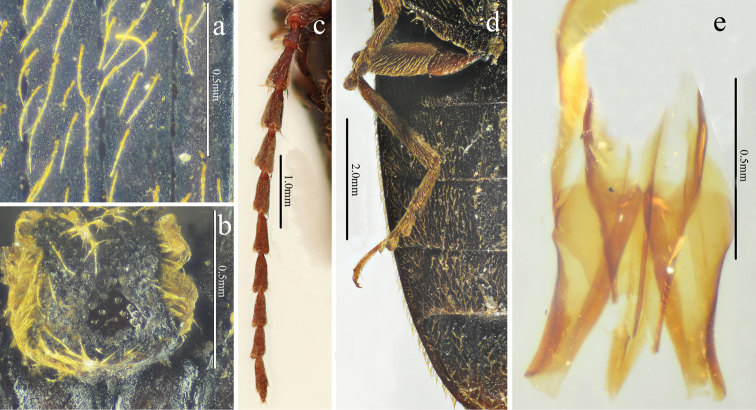
*Scutellathousquadrata* sp. nov., holotype, male **a** surface of elytra, dorsal view **b** scutellar shield, dorsal view **c** antenna, lateral view **d** hind leg and abdomen, ventral view **e** aedeagus, dorsal view.

**Figure 12.**
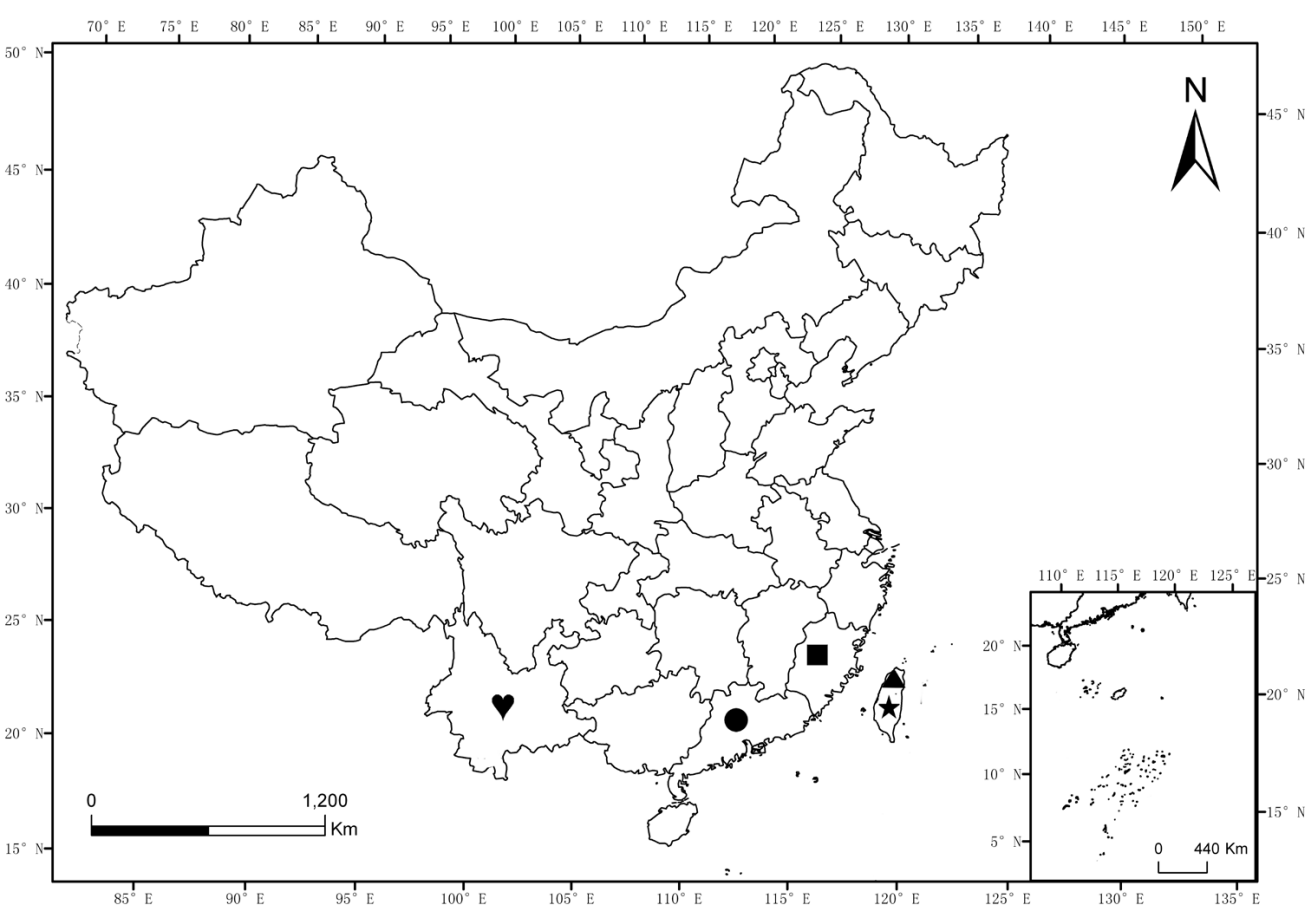
Distribution map of *Scutellathous* Kishii, 1955 in China. (triangle) *S.spinosus* Platia & Schimmel, 2007; (square) *S.quadrata* Liu & Jiang, sp. nov.; (heart) *S.habenularis* Liu & Jiang, sp. nov.; (circle): *S.nanlingensis* Liu & Jiang, sp. nov.; (star) *S.yamashitai* Arimoto, 1992.

Figs 9 , 10 , 11 , 12

#### Type locality.

Tianmu (Qianmutian) Mountains, Zhejiang, China.

#### Material examined.

Holotype: ♂ (SZPT), labels: 1) Zhejiang Prov., Tianmu (Qianmutian) Mts (1535 m, 30°23'37.85"N 119°26'25.85"E) (天目山), 2013.VII.1, leg. Jun Xu; 2) Holotype, *Scutellathousquadrata* sp.nov., Liu et al. 2019; 3) No. 20180356. Paratype: ♂ (SZPT), labels: 1) Zhejiang Prov., Tianmu (Qianmutian) Mts (1535 m, 30°23'37.85"N 119°26'25.85"E) (天目山), 2013.VII.1, leg. Mei Qin; 2) Paratype, *Scutellathousquadrata* sp.nov., Liu et al. 2019; 3) No. 20180358.

#### Diagnosis.

Body brown-black, dorsal pubescence partially hiding integument; anterior edge of head arched anterior-laterally in dorsal view; antennae reaching apices of pronotal hind angles, antennomere attached apico-dorsally near base, more centrally near apex, antennomere 3 2.2 times longer than 2 and 1.1 times longer than 4; pronotum with hind angles convergent posterad, disc with median non-furrowed glabrous line, with simple punctures; scutellar shield as wide as long, anterior edge straight, widest and rounded posteriorly, concave at sides.

*Scutellathousquadrata* is similar to *S.spinosus* Platia & Schimmel, 2007 (see Schimmel 2007) in body shape and size, but can be separated from the latter by the square-shaped scutellar shield (in *S.spinosus* it is 1.2 times longer than wide, measured from original figure), base without spine near humeral angles of the elytra (with spine in *S.spinosus*), the wholly brown-black body (in *S.spinosus* the body is entirely ferruginous), and the shape of parameres.

#### Description.

Male (holotype). Body (Fig. 9a–c) length 15.7 mm, body width 4.3 mm; brown-black, elytra (red-brown apically) and hypomera brown; antennae and legs brown-black, except tibiae brown; ventral side brown-black; dorsal pubescence pale, semi-recumbant, dense and pointed anterad on pronotum and head, nearly recumbent and pointed posterad on elytra, ventral pubescence more recumbent, longer and denser.

*Head.* Anterior edge arched anterior-laterally in dorsal view (Fig. 10a), spaces between punctures matt and less than 1 puncture diameter wide, punctures umbillicate and coarser, slightly denser in shallow triangular shallow depression; eyes semi-spherical, ocular index 75; last segment of maxillary palpus 1.5 longer than wide; antennae (Fig. 11c) reaching apices of pronotal hind angles, antennomeres 3 to 10 weakly serrate, attached apico-dorsally near base, more centrally near apex, antennomere 2 obconic, 1.4 times longer than wide, antennomere 11 oblong, 4.8 times longer than wide, proportions of antennomeres as follows: 100; 45; 99; 95; 101; 90; 90; 89; 79; 71; 100.

*Thorax.* Pronotum (Fig. 10c) 1.1 times longer than wide; parallel-sided in dorsal view, except slightly arched at anterior one-third and slightly widened before hind angles, widest just before apices of hind angles; disc moderately convex, with weak median non-furrowed glabrous line, hardly visible anteriorly; punctures simple, interspaces 2 to 3 puncture diameters wide medially, punctures larger and weakly umbillicate with interspaces 0.5 to 1 puncture diameter wide laterally and posteriorly; hypomera with spaces between punctures 1 puncture diameter wide; hind angles convergent posterad, apex short and obtuse, carina reaching anterad to basal third of pronotum along sides; sublateral incisions small, tooth-like. Prosternum (Fig. 10d) densely punctate, interspaces 1 to 2 times puncture diameters wide; anterior lobe 2.7 times wider than long, with sparse fine punctures. Prosternal process gradually concave behind procoxae and obtusely pointed at apex. Meso- and meta-ventrites with larger, denser punctures than on prosternum. Metaventrite furrowed medially throughout length.

*Scutellar shield.* (Fig. 11b) Length equal to width, straight anteriorly, widest and rounded posteriorly, concave at sides; disc convex with umbillicate punctures, spaces between punctures 2–3 puncture diameters wide medially, rugose-punctate with thick, outwardly-oriented pubescence near edges.

*Elytra.* Together 2.4 times longer than wide (Fig. 9a), 2.6 times longer and 1.1 times wider than prothorax, parallel-sided, gradually narrowing to apex from apical one-third, with punctate striae, strial punctures isodiametric, shallower toward apex; interstriae elevated basally, flat apically, with fine punctures, the interpuncture spaces about 2–3 puncture diameters wide (Figs 9a, 11a).

*Legs.* Tarsomere 3 with a longer lobe than tarsomere 2 (Fig. 11d), tarsomere 1 nearly as long as the following 3 tarsomeres together and 1.4 times longer than 5, tarsomere 4 shortest; metacoxal plate (Fig. 11d) gradually narrowed laterally, triangularly emarginate basally (tooth-like).

*Abdomen.* Surface of sternites III–VII like metaventrite, with punctures moreregular and dense and recumbent pubescence, interspaces with satin-like metallic sheen (Fig. 11d); sternite VII semicircular, 1.5 times wider than long, punctures evenly distributed.

*Genitalia.* Aedeagus (Fig. 11e, damaged) with penis gradually narrowing to pointed apex; parameres sinuate laterally near midlength, apical part nearly parallel-sided, apex truncate.

**Female.** Unknown.

**Variability.** Body length 14.7–15.7 mm; body width 3.7–4.3 mm.

**Larva.** Unknown.

#### Etymology.

The name of the new species refers to its quadrate scutellar shield.

#### Distribution.

China: Zhejiang (Tianmu Mts) (Fig. 12).

#### Biology.

Unknown.

#### Remarks.

Unfortunately, aedeagi of both specimens were damaged by poor preservation after prior dissection.

### Scutellathous spinosus

Taxon classificationAnimaliaColeopteraElateridae

Platia & Schimmel, 2007

Fig. 12

Scutellathous spinosus Platia & Schimmel, 2007: 59.

#### Diagnosis.

Following Platia and Schimmel (2007): length 13–16 mm, width 3.2–4 mm; red-brown with vague brown-black areas on head and pronotum; frons deeply impressed medio-anteriorly; antennae falling short of pronotal hind angles by about one antennomere, feebly serrate from antennomere 3, antennomere 2 more than twice longer than wide, antennomere 3 subtriangular, 2.5 times longer than 2, and longer than following; pronotum slightly longer than broad, widest at hind angles, disc strongly convex, abruptly sloping posterad, where with a trace of short median furrow, hind angles truncate, not divergent, with a short carina following edge, punctation coarse and variable; scutellar shield 1.2 times longer than wide, gently convex, sparsely punctate; elytra as broad pronotal posterior, 2.5–2.6 times longer than pronotum, base near humeral angles with a short spine.

Distinguished from other *Scutellathous* in China by elytral spine and red-brown colour. Distinguished from *S.yamashitai* by the shorter antennae not reaching the apices of the hind angles of the pronotum and rounded pronotal anterior angles.

#### Remarks.

No additions to the male genitalia except the length (1.75 mm) in original description. But we can see from the original image that the penis is reaching a little beyond the apex of the parameres, gradually narrowed to a pointed apex and the parameres are thick, strongly sinuate laterally at midlength, weakly tapered to a blunt apex.

#### Distribution.

China (Taiwan) (Fig. 12).

### Scutellathous yamashitai

Taxon classificationAnimaliaColeopteraElateridae

Arimoto, 1992

Fig. 12

Scutellathous yamashitai Arimoto, 1992: 73.

#### Diagnosis

(after Arimoto 1992). Length 12.6 mm, width 2.8 mm; body almost parallel-sided, flattened and shining dorsally, dark brown; antennae extending beyond apices of hind angles of pronotum by at least apical two antennomeres, antennomere 2 obconic, slightly longer than wide, antennomere 3 elongate triangular, about twice as long as 2 and longer than 4; pronotum as wide as long, with a shallow median impression in basal half, surface smooth and shining, sparsely and evenly punctate, hind angles short, divergent posterad, with carina; scutellar shield subvertical, with the sides somewhat constricted at posterior fourth; elytra about 2.9 times as long as humeral width, striae with coarse, uneven and elongate punctures, interstriae slightly elevated, irregularly punctate and transversely rugose; aedeagus with penis not reaching apex of parameres, apex of parameres depressed and furnished with some short setae.

*Scutellathousyamashitai* is similar to *S.comes* from Japan, but distinguished from by its darker color, smaller pronotal punctures, and divergent pronotal hind angles (Arimoto 1992).

#### Remarks.

No specimen was available for this study.

#### Distribution.

China (Taiwan) (Fig. 12).

## Supplementary Material

XML Treatment for Scutellathous

XML Treatment for Scutellathous habenularis

XML Treatment for Scutellathous nanlingensis

XML Treatment for Scutellathous quadrata

XML Treatment for Scutellathous spinosus

XML Treatment for Scutellathous yamashitai
